# Evaluation of country preparedness for Nipah and avian influenza zoonotic viral threats in Bangladesh

**DOI:** 10.1016/j.onehlt.2025.101075

**Published:** 2025-05-17

**Authors:** Md Mustafizur Rahman, Tristan Burgess, Jeffrey C. Mariner, Elizabeth Gold, Jonathon D. Gass

**Affiliations:** a Infectious Diseases Division, icddr,b (International Centre for Diarrhoeal Disease Research, Bangladesh), Dhaka, Bangladesh; b Center for Wildlife Studies, South Freeport, ME, USA; c Department of Infectious Disease and Global Health, Cummings School of Veterinary Medicine, Tufts University, North Grafton, MA, USA; d JSI Research and Training Institute, Inc., Arlington, VA, USA; e Department of Public Health and Community Medicine, School of Medicine, Tufts University, Boston, MA, USA

**Keywords:** Nipah virus, Avian influenza, One Health, Zoonoses, Bangladesh, Disease outbreaks, Pandemic preparedness

## Abstract

Nipah and avian influenza viruses (NiV and AIV) are priority zoonotic pathogens in Bangladesh and are also important globally because of their pandemic potential. To understand current strengths, areas for improvement, and opportunities to enhance preparedness for NiV and AIV in Bangladesh, as part of the USAID STOP Spillover program, 47 relevant stakeholders were surveyed, and two country leads of the primary surveillance systems were interviewed. Data was collected focusing on four different areas: research projects, surveillance systems, laboratories, and outbreak risk management systems. Despite progress in recent years, our study reveals significant gaps in preparedness, detection, and response capacities across sectors and highlights the importance of prioritizing extended and targeted surveillance, biosafety solutions for laboratories, and early event detection for resilient defenses against these viruses using a One Health framework.

## Highlights

• Systematic and routine monitoring antigenic drift and shift of AIV in wild birds and poultry is needed.
• Community-based surveillance is key for improving NiV case detection.
• Integrated surveillance across One Health sectors is required.
• Lab upgrades to BSL-3 are needed for enhanced biosafety and diagnostics.

## 1 Introduction

Bangladesh is a critical hotspot for zoonotic pathogen spillover due to dense human and diverse wildlife populations, rapid urbanization and deforestation, and climate change vulnerability [1]. Nipah virus (NiV) and avian influenza viruses (AIV) are considered priority zoonotic pathogens of concern for Bangladesh [2]. Since 2001, NiV has caused 244 deaths out of 343 human cases [3], while AIV has led to 1 death from 11 human cases and over 550 poultry outbreaks since 2007 [4,5]. Although NiV spillovers are infrequent [6] and AIV is endemic in poultry and domesticated waterfowl in Bangladesh [7], both pathogens have the potential to spawn the next pandemic [8,9].

The USAID STOP Spillover program conducted this study in which stakeholders from various government and non-government institutions working on NiV and AIV were engaged with the objective of understanding current strengths, areas for improvement, and opportunities to enhance national preparedness for NiV and AIV in Bangladesh.

## 2 Materials and methods

From January 2022 to May 2023, 47 interviews using structured questionnaires were conducted with stakeholders from government, research organizations, and academia concerning readiness for NiV and AIV threats from a One Health lens. We developed an initial list of stakeholders using our professional networks and organizational directories. Later, we applied the snowball method and gathered additional contacts from recruited participants. Moreover, two in-depth interviews were conducted using semi-structured questionnaires with country leads of the two primary surveillance systems for NiV and AIV in Bangladesh. Most of the interviews were face-to-face, although some were conducted virtually when in-person interviews were not possible. Data was collected in four different areas: research projects, surveillance systems, laboratories, and outbreak risk management systems. This represented a subset of ongoing and completed initiatives, and we included only available participants who provided consent to participate in the study.

### 2.1 Research efforts and addressing knowledge gaps

While we found six research studies on NiV and 16 on AIV, there was a notable lack of studies on wildlife for both pathogens. Among six identified research projects on NiV, only two collected biological samples to understand transmission dynamics, and the rest focused on date palm sap consumption behavior. The absence of comprehensive virological research on NiV limits understanding of spillover mechanisms from bats and variations in morbidity and mortality rates between primary and secondary human cases. Understanding community attitudes toward NiV is also crucial to understanding whether stigma causes people to avoid seeking healthcare, as it does with other communicable diseases. The case fatality rate for NiV in Bangladesh is significantly higher compared to other countries where Nipah outbreaks have occurred [10], which necessitates exploration of biological factors such as host heterogeneity and virus-specific variability. Although we found substantial research on AIV, the reasons behind the historically low incidence of detected human cases, despite high year-round prevalence in poultry, are still unclear. The comparative importance to humans of endemic poultry transmission versus new spillover events from wild birds remains unclear. A better understanding of transmission dynamics at this interface could also be gained through targeted research that samples wild birds and nearby poultry to compare strain similarities and estimate spillover frequency.

### 2.2 Surveillance capacity and expanding focus

We collected data on three surveillance systems on NiV. Established in 2006, hospital-based NiV sentinel surveillance in Bangladesh enables early case detection and rapid outbreak response. This system appears to be effective in identifying cases at ten participating hospitals, crucial for preventing secondary transmission, and monitoring changes in case frequency and spatial and temporal distribution. The system does not extend to cover the community-level adequately, which means many cases are likely missed, and this limits the scope to characterize spillover risks. Extended surveillance targeting animals (flying foxes) and testing forest products (primarily date palm sap) in outbreak areas would also enhance the capacity to identify and characterize spillover risk. Expanded Nipah virus surveillance would be costly, and the potential benefits have not been quantified. A pilot-scale implementation of fully integrated surveillance encompassing human, animal, and environmental sectors using the One Health framework could permit a cost-benefit analysis to inform decision-making on the value of such an expansion of the surveillance system.

Data on a total of 11 surveillance studies concerning AIV were collected. The AIV surveillance systems are among the most highly developed animal health surveillance systems in Bangladesh and are primarily conducted through ‘Sink Surveillance’ of the Department of Livestock Services (DLS), in collaboration with FAO ECTAD, in live bird markets of Dhaka and Chattogram city corporation areas. This system collects environmental samples and applies a source-sink model to predict pathogen accumulation. Eliminating monitoring needs at source farms and enabling pathogen detection in source at LBMs is a cost-effective way to run surveillance, which provides valuable data on transmission trends by assessing the prevalence and diversity of circulating AIV strains in LBMs. However, this system suffers from poor spatial resolution and limitations in terms of source identification. Since 2007, icddr,b, with funding from the US CDC, has led another initiative to identify circulating AIV strains in domestic poultry and LBM environments across five districts. While these efforts provide extensive surveillance, significant gaps exist since neither system currently monitors commercial or backyard poultry farms. We have also identified two hospital-based influenza surveillance systems, as well as two additional systems: one that monitors patients with febrile illnesses and another that tracks those with respiratory illnesses. While these four systems screen AIV in humans among other pathogens, there is currently no surveillance targeting high-risk populations such as LBM workers and farm workers. Poorly understood transmission dynamics, unknown importance of endemic poultry circulation versus wild bird introductions, and limited data integration highlight critical gaps. A sustained, large-scale surveillance of wild birds and in-contact poultry, including free-ranging ducks, will provide valuable information on AIV transmission dynamics, strain similarities, and spillover frequency, which will, in turn, aid in designing future interventions. Considering the identified gaps, monitoring for antigenic drift and shift in wild birds and poultry, and community-based surveillance targeting high-risk populations is also warranted. Effective human and animal data integration and securing sustainable funding for surveillance of both pathogens are necessary.

### 2.3 Laboratory capacity and diagnostic readiness

Of the eight laboratories identified, six had PCR analysis facilities for AIV, one for NiV, and one for both pathogens. Six laboratories reported the ability to analyze AIV samples, while only two could test for NiV (Table 1). None have BSL-3 facilities, limiting safe and effective research. IEDCR, the responsible government organization for infectious disease control and research among the human population, tests human samples for NiV and AIV. Similarly, DLS, responsible for animal disease control, tests AIV samples from poultry. However, the Bangladesh Forest Department (BFD), which is mandated to deal with disease outbreaks in wildlife [11], lacks pathogen detection capacity and uses its RT-PCR facility only for DNA barcoding for species identification.

**Table 1:** Laboratory capacity data in Bangladesh (data updated as of May 2023).

Pathogen	Laboratory *Host institution*	BSL Status	Testing capacity *Highest usage*
NiV	**BSL-2 Nipah Laboratory** *Institute of Epidemiology Disease Control and Research (IEDCR)*	**BSL-2**	**90/day** *Not testing now*
AIV	**BSL-2 Molecular Laboratory** *IEDCR*	**BSL-2**	**500/day** *200/day*
	**Central Disease Investigation Laboratory** *DLS (Department of Livestock Services)*	**BSL-2**	**100–200/day** *100–200/day*
	**National Reference Laboratory for AI** *Bangladesh Livestock Research Institute (BLRI)*	**BSL-2+**	**>750/day** *>500/day*
	**Department of Microbiology and Hygiene** *Bangladesh Agricultural University (BAU)*	**BSL-1**	**>96/day** *>96/day*
	**Department of Pathology** *BAU*	**BSL-2**	**200/day** *96/day*
	**Poultry Research and Training Centre** *Chattogram Veterinary and Animal Sciences University (CVASU)*	**BSL-1**	**92/day** *92/day*
NiV and AIV	**Virology Laboratory** *icddr,b*	**BSL-2+**	**500/day** *300/day*

All laboratories, except those affiliated with universities, reported participating in Proficiency Testing (PT) for external quality control. This involves comparing their results with unknown samples against those of other labs. In contrast, the three university labs reported relying solely on internal oversight or having no formal quality control system.

The biosafety level of these laboratories was not in compliance with the global standards for AIV isolation. BSL-2 facilities are required for detecting HPAI using nucleic acid amplification techniques like RT-PCR [12]. However, two laboratories are doing RT-PCR with only BSL-1 facilities. Moreover, HPAI should only be cultured under BSL-3 conditions [12,13], but BSL-2+ was the highest reported biosafety level in these laboratories, which is not a recognized containment level and lacks standardized operational guidelines, and such laboratories are also unsafe for airborne infectious pathogens [14]. As inadequate biosecurity may contribute to laboratory-acquired infections and environmental contamination, laboratories should be assessed for biosafety risk, obtain appropriate biosafety certifications, and comply with global guidelines for virus isolation. In the absence of BSL-3 laboratories and due to budgetary uncertainties, national stakeholders should prioritize human, non-human animal, and environmental health when considering AIV testing procedures.

### 2.4 Outbreak risk management and strategic preparedness

In Bangladesh, IEDCR and DLS lead disease control efforts in human and livestock, respectively, and manage outbreaks of NiV and AIV among other diseases. They work in collaboration with other government bodies, research organizations such as icddr,b, EcoHealth Alliance and development partners like US CDC and FAO ECTAD. However, despite these joint efforts in outbreak risk management, the limited scope of community-level surveillance and excessive dependence on media-based surveillance often result in delayed event detection and response, as well as potential underreporting and missed cases. In addition, the lack of integration of data across human, animal, and environmental health sectors affects identification of source and transmission route of outbreaks, which can be solved by regular and coordinated data-sharing efforts across sectors.

## 3 Conclusion

Our study highlights the urgent need for improving preparedness against priority zoonotic pathogens – NiV and AIV. Despite recent progress, critical gaps persist, such as limited surveillance coverage and inadequate virologic research capacity for a deeper understanding of spillover risks and transmission pathways. Biosafety challenges in laboratories demand collaboration and innovative solutions until BSL-3 facilities are established to ensure outbreak risk management capacity for rapid identification and control of outbreaks. Improvements in surveillance coverage, early detection, and data integration are imperative. Bridging these gaps requires coordinated efforts involving national and international stakeholders, improving Bangladesh's pandemic preparedness, and contributing to global resilience against emerging infectious diseases.

## CRediT authorship contribution statement

**Md Mustafizur Rahman:** Writing – review & editing, Writing – original draft, Project administration, Investigation, Formal analysis, Data curation. **Tristan Burgess:** Writing – review & editing, Writing – original draft, Methodology, Investigation, Formal analysis, Conceptualization. **Jeffrey C. Mariner:** Writing – review & editing, Methodology, Investigation, Conceptualization. **Elizabeth Gold:** Writing – review & editing, Methodology, Investigation. **Jonathon D. Gass:** Writing – review & editing, Writing – original draft, Supervision, Methodology, Investigation, Funding acquisition, Formal analysis, Conceptualization.

## Ethical approval

The Tufts Health Sciences IRB and icddr,b IRB determined that this study does not involve human subjects' research and does not require full IRB review. Nevertheless, as per IRB guidance, written informed consents were obtained from participants.

## Funding

This study was implemented by icddr,b and STOP Spillover Consortium and was funded by 10.13039/100000200USAID through 10.13039/100008090Tufts University (7200AA20CA00032). icddr,b is thankful to the core donors (governments of Bangladesh and Canada) for their ongoing support to icddr,b.

## Declaration of competing interest

The authors declare that they have no known competing financial interests or personal relationships that could have appeared to influence the work reported in this paper.

## Data Availability

The authors do not have permission to share data.
